# Unmet needs for treatment in 102 individuals with brief and limited intermittent psychotic symptoms (BLIPS): implications for current clinical recommendations

**DOI:** 10.1017/S2045796019000635

**Published:** 2019-11-19

**Authors:** Paolo Fusar-Poli, Andrea De Micheli, Monica Chalambrides, Aoife Singh, Castagnini Augusto, Philip McGuire

**Affiliations:** 1 Early Psychosis: Interventions and Clinical-detection (EPIC) Lab, Department of Psychosis Studies, Institute of Psychiatry, Psychology & Neuroscience, King's College London, London, UK; 2 OASIS Service, South London and Maudsley NHS Foundation Trust, London, UK; 3 Department of Brain and Behavioral Sciences, University of Pavia, Pavia, Italy; 4 National Institute for Health Research, Maudsley Biomedical Research Centre, South London and Maudsley NHS Foundation Trust, London, UK; 5 School of Child Neuropsychiatry, University of Modena and Reggio Emilia, Modena, Italy; 6 Department of Psychosis Studies, Institute of Psychiatry, Psychology & Neuroscience, King's College London, London, UK

**Keywords:** BLIPS, brief psychotic episode, prognosis, psychosis, risk, schizophrenia, prevention

## Abstract

**Aims:**

To investigate clinical outcomes and unmet needs in individuals at Clinical High Risk for Psychosis presenting with Brief and Limited Intermittent Psychotic Symptoms (BLIPS).

**Methods:**

Prospective naturalistic long-term (up to 9 years) cohort study in individuals meeting BLIPS criteria at the Outreach And Support In South-London (OASIS) up to April 2016. Baseline sociodemographic and clinical characteristics, specific BLIPS features, preventive treatments received and clinical outcomes (psychotic and non-psychotic) were measured. Analyses included Kaplan Meier survival estimates and Cox regression methods.

**Results:**

One hundred and two BLIPS individuals were followed up to 9 years. Across BLIPS cases, 35% had an abrupt onset; 32% were associated with acute stress, 45% with lifetime trauma and 20% with concurrent illicit substance use. The vast majority (80%) of BLIPS individuals, despite being systematically offered cognitive behavioural therapy for psychosis, did not fully engage with it and did not receive the minimum effective dose. Only 3% of BLIPS individuals received the appropriate dose of cognitive behavioural therapy. At 4-year follow-up, 52% of the BLIPS individuals developed a psychotic disorder, 34% were admitted to hospital and 16% received a compulsory admission. At 3-year follow-up, 52% of them received an antipsychotic treatment; at 4-year follow-up, 26% of them received an antidepressant treatment. The presence of seriously disorganising and dangerous features was a strong poor prognostic factor.

**Conclusions:**

BLIPS individuals display severe clinical outcomes beyond their very high risk of developing psychosis and show poor compliance with preventive cognitive behavioural therapy. BLIPS individuals have severe needs for treatment that are not met by current preventive strategies.

## Introduction

Individuals at Clinical High Risk for Psychosis (CHR-P hereafter (Fusar-Poli, 2017)) are detected by specialised clinical services through established psychometric assessment tools (Fusar-Poli *et al*., 2015*a*), in the context of a clinical interview (Fusar-Poli *et al*., 2017*c*). These tools assess whether the individual is meeting at least one of the three CHR-P subgroups: Attenuated Psychotic Symptoms (APS, about 85% of cases), Genetic Risk and Deterioration Syndrome (GRD, 5% of cases) or Brief and Limited Intermittent Psychotic Symptoms (BLIPS, 10% of cases) (Yung *et al*., 2005; Fusar-Poli *et al*., 2016*b*). During their recruitment (Fusar-Poli *et al*., 2016*g*), CHR-P individuals accumulate several risk factors for psychosis (Fusar-Poli *et al*., 2017*f*) and therefore have an enhanced risk for developing it. Their risk peaks during the first 2–3 years (Kempton *et al*., 2015) and is associated with the emergence of psychotic but not with non-psychotic disorders (Webb *et al*., 2015; Fusar-Poli *et al*., 2017*e*). Although the overall risk of the CHR-P group is 20% at 2 years (for details see eTable 4 in Fusar-Poli *et al*. (2016*b*)), this level of risk depends on the type of the CHR-P subgroups (Fusar-Poli *et al*., 2016*b*). The BLIPS subgroup, which is characterised by a short-lived and self-remitting psychotic episode, is associated with a very high risk of developing psychosis, with about one in two individuals developing psychosis over 2–3 years follow-up (Fusar-Poli *et al*., 2016*b*). This risk is higher than that observed in the APS or GRD subgroups (Fusar-Poli *et al*., 2016*b*). There are also some differences in the way the BLIPS is operationalised across CHR-P tools (for a comparative analysis see Fusar-Poli *et al*. (2016*d*)). For example, while the duration of the short-lived psychotic episode is <7 days under the Comprehensive Assessment of the At Risk Mental States (CAARMS (Yung *et al*., 2005)), it extends to 1 month under the Structured Interview for Psychosis-risk Syndromes (SIPS (McGlashan *et al*., 2010)) (for details see Table 1 in Fusar-Poli *et al*. (2016*a*)). Despite these differences, the prognosis of the two BLIPS operationalisations is comparable (Fusar-Poli *et al*., 2016*a*). A similar diagnostic and prognostic overlap is observed between the BLIPS designation and the standard DSM or ICD categories of Brief Psychotic Disorders and Acute and Transient Psychotic Disorders (ATPD (Fusar-Poli *et al*., 2016*a*, 2017*a*)). Since the DSM or ICD categories are more widely used by clinicians, there is a large window of a missed opportunity for extending preventive approaches in clinical routine (Rutigliano *et al*., 2018). However, in a recent study, we have demonstrated that these potentials are unexploited (Minichino *et al*., 2018). The first step to filling this gap is to characterise the broader clinical outcomes in the BLIPS and to address potential unmet needs for treatment. For example, because of the limited research in the BLIPS group, nothing is known beyond their risk of developing psychosis: their risk of being prescribed medications, being admitted to the hospital or receiving a Mental Health Assessment (MHA) informal admission. Furthermore, the uptake of NICE-recommended treatment for CHR-P individuals (Cognitive Behavioural Therapy, CBT (NICE, 2014)) in BLIPS individuals is unknown. Lastly, clinical prediction research into this field is scarce, likely because of the difficulty in identifying this infrequent subgroup. Previous empirical research has suggested that the duration of the short-lived psychotic episode, its abrupt onset, the presence of associated acute stress, the concurrent use of illicit substances or the presence of seriously disorganising or dangerous features may be significant predictors of outcomes in this population (Castagnini and Fusar-Poli, 2017; Fusar-Poli *et al*., 2017*a*).

**Table 1.** Clinical and sociodemographic characteristics of the sample

	*N*	Mean	s.d.
Age (years)	102	24.82	5.81
Baseline SOFAS	83	55.80	15.33
H0NOS (adjusted total)	66	9.66	6.36
		Median	IQR
CAARMS P1 severity			
P1 severity	79	6.00	6–6
P2 severity	79	6.00	6–6
P3 severity	78	5.00	1–5
P4 severity	78	2.00	0–4
	*N*	Count	%
Gender	102		
Females		45	44.12
Males		57	55.88
Borough	99		
Lambeth		47	47.47
Southwark		33	33.33
Lewisham		11	11.11
Croydon		8	8.08
Ethnicity	102		
White		44	43.14
Black		47	46.08
Other		11	10.78
Marital status	99		
Married		4	4.04
Separated or divorced		3	3.03
Single		78	78.79
In a relationship		14	14.14
Employment status	100		
Employed		34	34.00
Student		32	32.00
Unemployed		34	34.00

This study aims at unravelling the broader clinical outcomes in individuals experiencing a BLIPS and highlighting their potential unmet needs. Firstly, we will describe the uptake of NICE-recommended treatment as well as other types of treatments in this population. Secondly, we will describe the risk of developing poor clinical outcomes such as the risk for the first admission to mental health hospital, of receiving a first compulsory treatment, of developing a psychotic disorder and of receiving a first antipsychotic or antidepressant treatment. Thirdly, we will describe the association between candidate prognostic predictors and clinical outcomes.

## Methods

### Sample

We included all CHR-P subjects referred for suspicion of psychosis risk to the Outreach and Support in South London (OASIS) service, South London and Maudsley (SLaM) NHS Foundation Trust (Fusar-Poli *et al*., 2013), who met a refined version of the BLIPS CAARMS 12/2006 criteria (Yung *et al*., 2006) (see below) up to April 2016. The OASIS team covers a catchment area of 1.36 million of individuals in South London (Lambeth, Southwark, Lewisham and Croydon boroughs), where there is one of the highest rates of psychosis in the world (Jongsma *et al*., 2018) and therefore a large proportion of BLIPS among CHR-P individuals (Fusar-Poli *et al*., 2016*b*).

### Design

Prospective naturalistic long-term (up to 9 years) cohort study in CHR-P subjects who met BLIPS criteria. The clinical assessment and follow-ups were done as part of the standard clinical routine of OASIS.

### Clinical assessment

#### CHR-P assessment

The details of the psychopathological CHR-P assessment conducted at OASIS have been described previously (Fusar-Poli *et al*., 2013). In brief, the CHR-P assessment is based on the CAARMS 12/2006 (Yung *et al*., 2005) which assesses APS, GRD and BLIPS. However, while in the original CAARMS 12/2006 criteria for the BLIPS group, the maximum duration of the short-lived psychotic episode is limited to 7 days, in this study, the duration of the BLIPS has been extended to 1 month. This was done in light of the similar prognostic outcome of short-lived psychotic episodes with different initial duration (Fusar-Poli *et al*., 2016*a*) and to improve the diagnostic consistency between the CHR paradigm and international manuals. Clinical follow-up is usually performed as part of the standard care and it is facilitated by the use of an Electronic Health Record (HER) that has been described elsewhere (Stewart *et al*., 2009) and already validated in this population (Fusar-Poli *et al*., 2016*e*, 2016*f*, 2017*d*, 2017*e*). The OASIS team offers focused interventions spanning pharmacological, psychological and psychoeducational activities for 2 years (Fusar-Poli *et al*., 2015*b*).

### Study measures

#### Baseline sociodemographic and clinical characteristics

Baseline descriptive variables included: age, gender, SLaM borough, ethnicity, marital status, employment status, HONOS (Health Of the Nation Outcome Scale HoNOS (Orrell *et al*., 1999) total score), Social and Occupational Functioning Assessment Scale (SOFAS) (Rybarczyk, 2011) and severity of APS (CAARMS P1-P4 (Yung *et al*., 2006)).

#### Specific baseline characteristics of the BLIPS

Specific characteristics of the BLIPS episodes included: duration of BLIPS episodes (in days), type of BLIPS (BLIPS alone or in combination with APS and/or GRD criteria), the presence of seriously disorganising and dangerous features (defined as previously detailed (Fusar-Poli *et al*., 2017*a*)), the type of onset, the presence of associated acute stress, life events, substance-induced BLIP, lifetime substance use, familial history of any mental disorder, trauma and comorbid borderline personality disorder. The criterion of seriously disorganising or dangerous symptom is formally scored in the SIPS manual. As previously observed (Fusar-Poli *et al*., 2017*a*), ‘dangerous’ is taken to mean physically dangerous, e.g. risk of death or serious physical injury, and ‘disorganising’ means potentially psychosocially dangerous, e.g. risk of seriously damaging work relations, social relations, family relations or personal dignity. The type of onset was defined in line with the ICD-10 specifier for ATPD: a change from a state without psychotic features to an abnormal psychotic state, within a period of 2 weeks or less (acute onset) or within 48 h (abrupt onset). Associated acute stress is also taken from the same ICD-10 specifier for ATPD to mean that the first psychotic symptoms occur within about 2 weeks of one or more events that would be regarded as stressful to most people in similar circumstances, within the culture of the person concerned (e.g. bereavement, unexpected loss of partner or job, marriage, or the psychological trauma of combat, terrorism and torture). In line with the ICD-10 specifications for ATPD, long-standing difficulties or problems experienced as stressful or threatening (e.g. medical comorbidity, childbirth, etc.) occurring within 2 weeks to 1 month before the symptom onset were not coded as associated acute stress but in the variable life events. The variable substance-induced BLIPS was operationalised through the CAARMS specifier pattern of symptoms, which notes that the symptoms emerge only in relation to substance use. The variable lifetime drug use coded any exposure to illicit substances before the onset of a BLIP. The familial history of mental disorder was defined as the presence of any non-organic mental disorders in the first- or second-degree relatives. The variable trauma included lifetime emotional, physical, sexual trauma and neglect, as recorded by clinicians on the EHR.

#### Treatments received by the BLIPS individuals over the follow-up

At OASIS all individuals are offered needs-based interventions and clinical monitoring for 2 years. Family interventions can also be offered by OASIS as part of needs-based interventions. In addition to this, all individuals are systematically offered the NICE-recommended first-line treatment for preventing the onset of psychosis (NICE, 2014), namely CBT for psychosis. Evidence indicates that the ‘minimum effective’ dose of CBT for psychosis is of 16 sessions (in line with the NICE recommendation) although 25 sessions are the ‘more appropriate’ dose (Lincoln *et al*., 2016). Accordingly, OASIS offers up to 25 sessions of CBT, with the additional option of boosting sessions if needed, over a period of 2 years. This study, therefore, operationalised the exposure to CBT in line with these recommendations.

At OASIS, Medications such as antidepressants, benzodiazepines or mood stabilisers can be added to CBT if the CHR-P individuals still display symptoms, functional impairment or distress. Antipsychotics are not generally used unless the CHR-P individual is about to transition to a frank episode of psychosis. Accordingly, exposure to antidepressants, benzodiazepines, mood stabilisers and antipsychotics was recorded. Additional variables included the time to discharge from OASIS, the number of clinical services that took care of the BLIPS individuals once they were discharged from the team and how many individuals developed psychosis after their discharge from OASIS. Treatments considered in this study are only those received during the CHR-P individuals during their care under OASIS.

#### Clinical outcomes in BLIPS

The primary outcome measure of the current study was the risk of developing poor clinical outcomes in BLIPS individuals. This was operationalised as the risk for the first admission to mental health hospital, the risk of receiving a first compulsory treatment through an MHA sectioning, the risk of developing a psychotic disorder, the risk of receiving a first antipsychotic treatment and the risk of receiving a first antidepressant treatment.

### Statistical analysis

Sociodemographic and clinical characteristics of the sample, as well as treatments uptake over follow-up, were described with mean and s.d. for continuous variables and absolute and relative frequencies for categorical variables. The primary outcome (clinical outcomes of BLIPS) was investigated plotting Kaplan Meier (Kaplan and Meier, 1958) failure functions (1-survival) (Kaplan and Meier, 1958) and Greenwood 95% CIs (Greenwood, 1926), summarising the cumulative risk of a first admission to mental health hospital, an MHA compulsory admission, developing a psychotic disorder, receiving a first antipsychotic treatment and receiving a first antidepressant treatment. For each outcome, we reported the yearly point estimates of the relative cumulative incidence, truncated when <10 individuals were still at risk. We explored the association between candidate predictors and poor clinical outcomes in BLIPS using Cox proportional hazards models, after checking for proportional hazards assumption (Grambsch and Therneau, 1994). Candidate factors were selected on the basis of *a priori* knowledge, as indicated in the introduction: duration of the BLIPS, type of onset, presence of associated acute stress, substance-induced BLIPS and seriously disorganising or dangerous features. For all the analyses, statistical tests were two-sided and statistical significance was defined as *p* values of <0.05. All analyses were conducted in STATA 14 (STATA Corp., TX, USA).

## Results

### Baseline sample characteristics

As shown in Tables 1 and 2, 102 subjects with BLIPS (56% males) attended the OASIS service until April 2016 across all SLaM boroughs (mostly Lambeth and Southwark). Their mean age was 25 years (s.d. = 5.81), 79% of them were single and 34% unemployed. The proportion of white (43%) and black (46%) ethnicities was similar (black individuals are significantly over-represented in OASIS compared to the background population (Byrne *et al*., 2019)). The baseline functional level was rather low (SOFAS = 56, s.d. = 15.33) and reflected by a HONOS score of 10 (s.d. = 6.36). Most BLIPS subjects (60%) did not meet additional CHR-P subgroups. About one-third (30%) of them displayed seriously disorganising or dangerous features. The BLIPS episodes lasted on average 7 days (range 1–30, 22 BLIPS had a duration between 7 and 30 days). In about one-third of the BLIPS (35%), the onset was abrupt (within 48 h). Acute stress was present in about one-third of BLPS (32%), while significant life events were noted in half of the cases (49%). A minority of the BLIPS episodes (20%) were induced by illicit substances, but the proportion of lifetime use of illicit substances was higher (55%). About 38% of individuals with BLIPS presented a positive familial history for psychotic or non-psychotic mental disorders. Lifetime trauma was recorded in about 45% of BLIPS, while the proportion of comorbid emotionally unstable personality disorder was 24%.

**Table 2.** Baseline specific characteristics of the BLIPS

	*N*	Mean	s.d.
BLIPS duration (days)	75	7.17	5.50
	*N*	Count	%
Type of onset	88		
Abrupt		31	35.23
Acute		57	64.77
Associated acute stress	94		
No		64	68.09
Yes		30	31.91
Life events	92		
No		47	51.09
Yes		45	48.91
Substance-induced BLIPS	94		
No		74	78.72
Yes		20	21.28
Lifetime substance use	94		
No		42	44.68
Yes		52	55.32
Familial history of any mental disorder	91		
No familial mental disorders		56	61.54
Familial history for non-psychotic mental disorders		20	21.98
Familial history for psychotic mental disorders		10	10.99
Familial history for psychotic and non-psychotic mental disorders		5	5.49
Lifetime trauma	85		
No		48	56.47
Yes		37	45.53
Comorbid Emotionally Unstable Personality Disorder	68		
No		52	76.47
Yes		16	23.53
BLIPS subgroup	102		
BLIPS only		61	59.80
BLIPS + APS		36	35.29
BLIPS + GRD		1	0.98
BLIPS + APS + GRD		4	3.92
BLIPS seriously disorganising and dangerous	97		
No		67	69.07
Yes		30	30.93

### Treatments received over the follow-up time

The vast majority of BLIPS individuals (80%), despite being systematically offered CBT for psychosis, did not fully engage with it and did not receive the minimum effective dose (Table 3). Only three BLIPS individuals out of 102 (2.94%) received the appropriate dose of CBT. The proportion of BLIPS individuals receiving antidepressant treatment was 42%. About 10% of them received benzodiazepines or promethazine, 3% received mood stabilisers and 2% a combination of these medications. The majority of BLIPS (69%) was prescribed antipsychotic medications over follow-up. The BLIPS individuals were discharged by OASIS on average after 489 days (1.34 years), and the majority of them (58%) eventually received the care from at least one mental health service. About one-third (27%) of BLIPS individuals who transitioned to psychosis become unwell after being discharged from OASIS.

**Table 3.** Treatments received by the BLIPS individuals over the follow-up

	*N*	Mean	s.d.
Time to discharge from OASIS (days)	95	488.86	500.72
	*N*	Count	%
Cognitive Behavioural Therapy	95		
None		42	44.21
Subeffective dose		34	35.79
Minimum effective dose^a^		19	20.00
Antidepressants	62		
No		36	58.06
Yes		26	41.94
Antipsychotics	67		
No		21	31.34
Yes		46	68.66
Other medications	96		
None		81	0.84
Benzodiazepines or promethazine		10	0.10
Mood stabilisers		3	0.03
Benzodiazepines, promethazine and mood stabilisers		2	0.02
Individuals developing psychosis after discharge from OASIS	37		
No		27	72.97
Yes		10	27.03
Number of clinical services after OASIS	93		
None		39	41.94
1		20	21.51
2		10	10.75
>2		24	25.80

a Three individuals received an ‘appropriate dose’ of CBT only.

### Clinical outcomes in individuals with BLIPS

#### Clinical outcomes

**Fig. 1.**
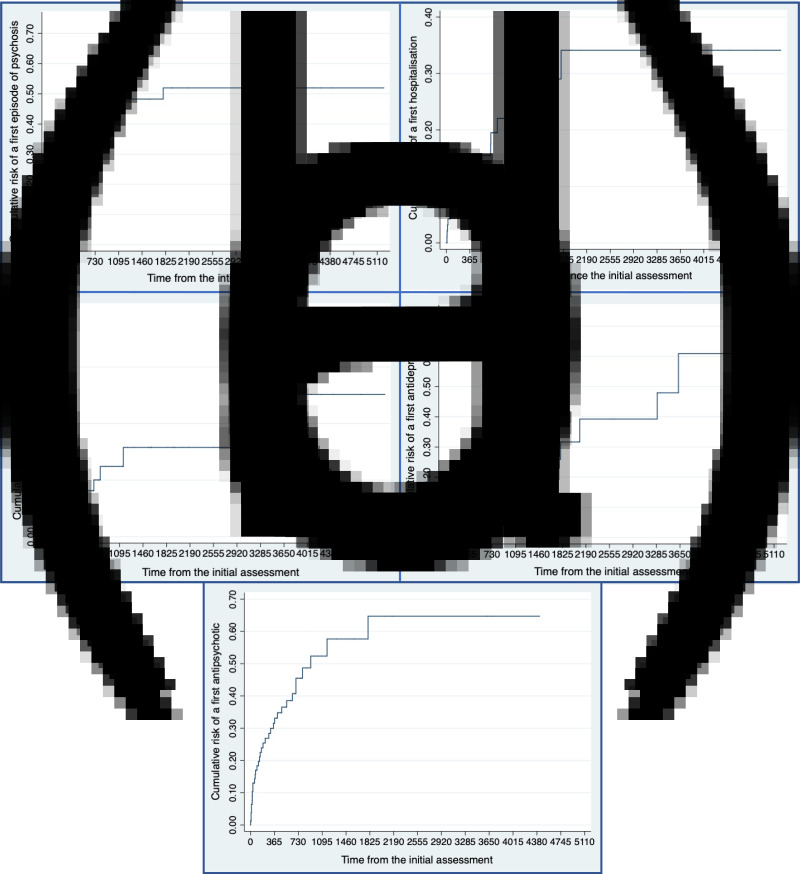
Clinical outcomes in 102 patients with a BLIPS over follow-up time (days). (a) Cumulative risk of a first episode of psychosis, (b) cumulative risk of a first hospitalisation, (c) cumulative risk of a first MHS section, (d) cumulative risk of a first antidepressant, (e) cumulative risk of a first antipsychotic.

The cumulative risk of a first episode of psychosis was 0.194 (95% CI 0.126–0.290) at 1 year (67 individuals still at risk), 0.299 (95% CI 0.211–0.415) at 2 years (40 individuals still at risk), 0.428 (95% CI 0.314–0.562) at 3 years (23 individuals still at risk), 0.483 (95% CI 0.359–0.624) at 4 years (17 individuals still a risk), 0.519 (95% CI 0.368–0.667) at 5 years (14 individuals still at risk) (Fig. 1*a*).

The cumulative risk of a first hospitalisation (Fig. 1*b*) was 0.124 (95% CI 0.071–0.214) at 1 year (68 individuals still at risk), 0.195 (95% CI 0.120–0.309) at 2 years (38 individuals still at risk), 0.253 (95% CI 0.157–0.391) at 3 years (25 individuals still at risk), 0.291 (0.181–0.445) at 4 years (19 individuals still at risk), 0.341 (95% CI 0.211–0.519) at 5 and 6 years (ten individuals still at risk).

The cumulative risk of a first MHA (Fig. 1*c*) was 0.067 (95% CI 0.031–0.144) at 1 year (73 individuals still at risk), 0.101 (95% CI 0.051–0.195) at 2 years (45 individuals still at risk), 0124 (95% CI 0.064–0.233) at 3 years (30 individuals still at risk), 0.158 (95% CI 0.082–0.292) at 4 years (24 individuals still at risk), 0.158 (95% CI 0.082–0.292) at 5–9 years (ten individuals still at risk).

The cumulative risk of a first antidepressant (Fig. 1*d*) was 0.173 (95% CI 0.1042–0.281) at 1 year (56 individuals still at risk), 0.226 (95% CI 0.144–0.345) at 2 and 3 years (24 individuals still at risk), 0.260 (95% CI 0.164–0.396) at 4 years (19 individuals at risk), 0.317 (95% CI 0.192–0.493) at 5 years (13 individuals still at risk).

The cumulative risk of a first antipsychotic (Fig. 1*e*) was 0.315 (95% CI 0.221–0.437) at 1 year (44 individuals still at risk), 0.455 (95% CI 0.338–0.589) at 2 years (22 individuals still at risk), 0.524 (95% CI 0.390–0.671) at 3 years (11 individuals still at risk).

### Factors associated with clinical outcomes in BLIPS individuals

The only prognostic factor which was significantly associated with the risk of psychosis onset, risk of first hospitalisation and risk of first MHA section was the presence of seriously disorganising and dangerous features (Table 4). This factor was not associated with the risk of first antipsychotic or the risk of the first antidepressant. The duration of BLIPS, abrupt onset, presence of associated acute stress and the presence of a substance-induced BLIPS were not associated with any clinical outcome.

**Table 4.** Factors associated with clinical outcomes in BLIPS

	First hospitalisation				First MHA section			
	HR	95%CI		*p*	HR	95% CI		*p*
Duration of BLIPS	1.092	0.2	1.013	1.176	1.075	0.983	1.175	0.116
Type of onset^a^	1.435	0.373	5.524	0.599	1.147	0.225	5.852	0.869
Presence of associated acute stress^b^	1.951	0.595	6.398	0.27	3.085	0.707	13.45	0.134
Substance-induced BLIPS^b^	NA	NA	NA	NA	NA	NA	NA	NA
Seriously disorganising and dangerous features^b^	4.325	1.258	14.871	0.02	8.505	1.687	42.87	0.009

	Psychosis onset				First antipsychotic				First antidepressant			
	HR	95%CI		*p*	HR	95% CI		*p*	HR	95%CI		*p*
Duration of BLIPS	1.071	0.991	1.158	0.081	1.005	0.937	1.078	0.882	0.908	0.756	1.092	0.307
Type of onset^a^	1.677	0.509	5.525	0.395	0.449	0.138	1.467	0.185	0.763	0.186	3.134	0.707
Presence of associated acute stress^b^	2.155	0.76	6.109	0.149	1.844	0.698	4.873	0.217	0.78	0.184	3.311	0.737
Substance-induced BLIPS^b^	1.28	0.266	6.175	0.758	1.779	0.469	6.751	0.397	NA	NA	NA	NA
Seriously disorganising and dangerous features^b^	3.117	1.082	8.978	0.035	2.424	0.883	6.654	0.086	1.468	0.377	5.71	0.58

a Abrupt *v.* acute as reference.

b Yes *v.* no as reference.

## Discussion

To our best knowledge, this is the largest cohort of BLIPS individuals (*n* = 102) with the longest follow-up (up to 9 years). They were mostly young males of black and white ethnicities with low baseline functioning. The onset of the BLIP was abrupt and associated with acute stress in one-third of the cases, and only in a minority of the cases with illicit substance use. The vast majority (80%) of BLIPS individuals, despite being systematically offered CBT for psychosis, did not engage with it and did not receive the minimum effective dose. Only 3% of BLIPS received the appropriate dose of CBT. At 4-year follow-up, 52% of the BLIPS developed a psychotic disorder, 34% were admitted to hospital and 16% received a compulsory admission. At 3-year follow-up, 52% of them received an antipsychotic treatment and 26% an antidepressant treatment. The presence of seriously disorganising and dangerous features was confirmed to be a significant prognostic factor. These findings indicate that BLIPS individuals have severe unmet needs for treatment that are not currently targeted by preventive interventions.

This study has several methodological strengths. Firstly, it is based on a difficult-to-run prospective study in a rare clinical phenotype. Secondly, it leverages of a large-scale database which is indexing real-world outcomes and with a long-term follow-up of up to 9 years. The outcomes investigated in our database are high in ecological validity (i.e. they represent real-world clinical practice). The combination of real-world ecological outcomes and psychometric interviews (e.g. CAARMS-based definition of psychosis onset) in a longitudinal design is rare in the CHR-P literature (Webb *et al*., 2015). Building on these methodological strengths, this study characterised for the first time the broad clinical outcomes of BLIPS individuals over time. These strengths represent substantial and distinctive advancements of knowledge compared to our previous publication in BLIPS individuals (Fusar-Poli *et al*., 2017*a*), which had a smaller sample size, shorter follow-up time and did not investigate clinical outcomes other than the risk of developing a first episode of psychosis, nor the uptake of preventive treatments over clinical care that is necessary to assess unmet treatment needs.

Firstly, this study described the uptake of preventive treatments that are recommended by current clinical guidelines. As noted above, CBT is the first-line treatment recommended not only by the NICE (NICE, 2014) but also by the EPA (Schmidt *et al*., 2015) guidelines, while antipsychotic treatment is highly discouraged. Although there is evidence indicating that these recommendations are broadly generalisable to clinical practice settings (Lincoln *et al*., 2016), no empirical study has ever tested it in BLIPS samples. This study demonstrated that only one in five BLIPS is receiving the minimum effective dose and only three in 100 is receiving the appropriate dose of CBT. Should these findings be replicated in other CHR-P sites, they question the meaningfulness of recommending CBT to prevent psychosis in these individuals. Although the subjective reasons for such a poor uptake were not directly investigated in this study, the current recommendations for the prevention of psychosis in CHR-P individuals are based on a one-size-fits-all approach, which is associated with uncertain efficacy of CBT in these individuals (Davies *et al*., 2018*a*, 2018*b*). This contrasts with the recent pressuring call for precision medicine treatments as well with the substantial clinical, diagnostic and prognostic heterogeneity of the CHR-P population. As a consequence of such a ‘shotgun approach’, protocols for CBT for psychosis prevention have been tailored to support the most frequent subgroup of CHR-P individuals presenting with APS. However, APS symptoms are not necessarily present in BLIPS individuals. Therefore, the extent to which current CBT for psychosis fits the BLIPS is unknown. Our clinical experience suggests that a substantial proportion of BLIPS individuals do not want to recall and travel through again their dramatic and acute experience of psychosis onset. Because their experience of psychosis has self-resolved, BLIPS individuals seem keener than other CHR-P subgroups to focus on maintaining their educational, professional or leisure achievements as opposed to engaging in a long course of talking therapy. Interventions that are targeting these needs and promoting good health as opposed to reduction of APS (that are not typically present in BLIPS cases) be better endorsed by BLIPS individuals. For example, there is converging evidence that CHR-P individuals display cardiometabolic risk factors which are largely modifiable (Carney *et al*., 2016, 2018). Accordingly, interventions aiming at improving the physical health of BLIPS individuals (Vancampfort *et al*., 2019) may, at the same time, support their engagement with the CHR-P services and reduce cardiometabolic risk factors. The latter point is particularly indicated in light of the BLIPS individuals' high likelihood of receiving antipsychotic treatments during the clinical care (see below), which are associated with the alterations of physical health outcomes (Srihari *et al*., 2015). According to our clinical experience, a relatively high proportion of BLIPS patients unilaterally decide to be discharged from the service, likely because they do not feel that the treatment offered is helpful. Overall, qualitative research is needed to elucidate the subjective preference as well as the reasons for premature discharge from OASIS, in order to inform the development of appropriate treatments that may be widely accepted by BLIPS individuals.

Secondly, this study described the risk of developing poor clinical outcomes. The very high risk of developing psychosis of BLIPS individuals is not a novel finding (Fusar-Poli *et al*., 2016*c*, 2017*a*). However, the current manuscript adds to this point by showing that about one-third of BLIPS individuals who transitioned to psychosis did so after being discharged from OASIS. Furthermore, BLIPS individuals were discharged by OASIS on average after 1.34 year, well ahead the 2-year recommended clinical follow-up. The additional novel finding is that half of the BLIPS (52% at 3 years) eventually received some antipsychotic treatment, which is not recommended by the current clinical guidelines (NICE, 2014; Schmidt *et al*., 2015). Therefore, not only the BLIPS do not engage with the recommended preventive treatments, but they also eventually end up in receiving the only treatments that are not recommended for their problems. Antidepressant exposure was modest, as well as the use of other medication, indicating that these individuals are not receiving any other systemic treatment for their problems. Because of the lack of an effective treatment which is widely accepted, most of the BLIPS (58%) are still in need of care after being discharged from OASIS. About one-third of these fragile individuals eventually are being admitted to a mental health hospital (34% at 4 years). This value parallels the proportion of ATPD individuals being admitted to mental health hospital over follow-up time (33% at 8 years (Minichino *et al*., 2018)). Whatever the label used to define short-lived psychotic episodes, the clinical outcome is similar, and CHR-P services do not substantially impact their course, compared to standard care. Accordingly, about 16% of BLIPS received a compulsory admission at 4 years. A recent population-based study in the same NHS Trust showed comparable rates of first-episode psychosis cases receiving compulsory admission (106/446 = 23.76%) (Oduola *et al*., 2019). These findings, taken altogether, clearly indicate that BLIPS individuals are at high risk of developing serious mental health outcomes beyond the development of psychosis and that these outcomes are not currently addressed by existing mental health services. At a minimum, more assertive monitoring strategies should be implemented for this population, along with specific relapse prevention programmes (Fusar-Poli *et al*., 2017*b*). Recent advancements in e-Health approaches for CHR-P individuals could allow an optimised monitoring of clinical outcomes in young populations and should be the subject of future research (Reilly *et al.*, 2019; Fusar-Poli *et al.*, 2019).

Thirdly, this study described the association between candidate prognostic predictors and clinical outcomes in BLIPS. Our group has previously demonstrated that the presence of seriously disorganising or dangerous features was a robust prognostic factor when controlling for several confounders including age, HONOS, SOFAS, CAARMS P1-P4 total score, gender, borough, ethnicity, marital status, employment status, BLIPS subgroup and recurrence of BLIPS (Fusar-Poli *et al*., 2017*a*). This study adds to the previous analysis by exploring in a multivariate approach the impact of new potential predictors. For example, this is the first study testing the actual impact of BLIPS episodes lasting <7 days or between 7 days and 1 month. Since there were no significant differences in any of the outcomes explored, the duration of the BLIPS could be extended to 1 month to better align with the operationalisation of the SIPS/SOPS and the DSM-5. The other predictors that were considered (abrupt onset, presence of associated acute stress and the presence of a substance-induced BLIPS) were not associated with any clinical outcomes. The only significant predictor of the risk of psychosis onset, risk of first hospitalisation and risk of first MHA section remained the presence of seriously disorganising or dangerous features. The poor outcomes associated with this predictor may reflect the presence of extreme state factors within the BLIPS subgroup, as initially hypothesised (Cornblatt and Carrion, 2015). Seriously disorganising or dangerous features have been imported from earlier works on psychopathological subtypes of schizophrenia, which indicated that a drift towards disorganisation (hebephrenia) links with ‘deterioration’ and worst functional outcome (McGlashan and Fenton, 1993) (see also Heckert's concept of hebephrenia (Sedler, 1985)). Overall, these findings corroborate the notion that investigating seriously disorganising or dangerous features should become standard practice when assessing CHR-P individuals.

There are also some limitations to this study. Although this is the largest cohort study in BLIPS individuals ever conducted, it was still representing a relatively small sample size. As such, the current study may have been underpowered to estimate the impact of substance-induced BLIPS on the first hospitalisation, first MHA section and first prescription of antidepressant. Because of these inherent limitations, we did not seek to develop a prognostic model and to validate it but rather provided descriptive analyses on the potential association of prognostic factors and clinical outcomes. To further contain the risk of fishing expeditions and type I false positives, we restricted the Cox regression analyses to a subset of *a priori* predictors that were selected based on *a priori* clinical knowledge, in line with methodological recommendations (Fusar-Poli *et al*., 2018). A further limitation is that the association between seriously disorganising or dangerous features needs to be replicated and confirmed in external and independent samples. A final limitation to this study is that the reasons for poor uptake of CBT in BLPS patients were not qualitatively explored.

## Conclusions

BLIPS individuals display severe clinical outcomes beyond their very high risk of developing psychosis. There is poor uptake of preventive CBT in BLIPS individuals. These individuals have severe needs for treatment that are not met by current preventive strategies.

## Data

There is no ethical permission for data sharing.
